# Effective connectivity of visual word recognition and homophone orthographic errors

**DOI:** 10.3389/fpsyg.2015.00640

**Published:** 2015-05-20

**Authors:** Joan Guàrdia-Olmos, Maribel Peró-Cebollero, Daniel Zarabozo-Hurtado, Andrés A. González-Garrido, Esteve Gudayol-Ferré

**Affiliations:** ^1^Facultat de Psicologia, Institut de Recerca en Cognició, Cervell i Conducta, Universitat de BarcelonaBarcelona, Spain; ^2^Department of Methodology of Behavioral Sciences, School of Psychology, University of BarcelonaBarcelona, Spain; ^3^Instituto de Neurociencias, Universidad de GuadalajaraGuadalajara, Mexico; ^4^Universidad Michoacana de San Nicolás de HidalgoMorelia, Mexico

**Keywords:** fMRI, orthography, homophone errors, reading, structural equation models, dynamic causal modeling

## Abstract

The study of orthographic errors in a transparent language like Spanish is an important topic in relation to writing acquisition. The development of neuroimaging techniques, particularly functional magnetic resonance imaging (fMRI), has enabled the study of such relationships between brain areas. The main objective of the present study was to explore the patterns of effective connectivity by processing pseudohomophone orthographic errors among subjects with high and low spelling skills. Two groups of 12 Mexican subjects each, matched by age, were formed based on their results in a series of *ad hoc* spelling-related out-scanner tests: a high spelling skills (HSSs) group and a low spelling skills (LSSs) group. During the *f* MRI session, two experimental tasks were applied (spelling recognition task and visuoperceptual recognition task). Regions of Interest and their signal values were obtained for both tasks. Based on these values, structural equation models (SEMs) were obtained for each group of spelling competence (HSS and LSS) and task through maximum likelihood estimation, and the model with the best fit was chosen in each case. Likewise, dynamic causal models (DCMs) were estimated for all the conditions across tasks and groups. The HSS group’s SEM results suggest that, in the spelling recognition task, the right middle temporal gyrus, and, to a lesser extent, the left parahippocampal gyrus receive most of the significant effects, whereas the DCM results in the visuoperceptual recognition task show less complex effects, but still congruent with the previous results, with an important role in several areas. In general, these results are consistent with the major findings in partial studies about linguistic activities but they are the first analyses of statistical effective brain connectivity in transparent languages.

## Introduction

Reading is a cognitive process that requires visually identifying written elements and their respective phonological association to form meaning. In recent years, the study of reading under a neurocognitive perspective has focused on reading disabilities, mainly accuracy and speed (Wolf et al., 2000; Leinonen et al., 2001; Torppa et al., 2007; Nation, 2008), as well as on their possible origin and their respective brain functioning. From an anatomical-functional perspective, the development of the skills needed to process the information of the orthographic structure, phonology and meaning of words, involves activating the participation of several brain regions, mainly cortical, thus forming what some authors have called a specialized system for reading (Berninger and Richards, 2002).

Some authors like Perfetti and Bolger (2004) or Hills et al. (2005) have proposed three main anatomical regions involved in this reading system: occipital-temporal, temporal-parietal, and inferior frontal. The first of them, which includes the fusiform and lingual gyruses, has been related to the structural or morphological (orthographic) analysis of words. Activations have been reported in the left hemisphere’s fusiform gyrus when faced with the presentation of words in tasks demanding relatively simple manipulation or processing, such as visual priming (Devlin et al., 2006; Glezer et al., 2009), lexical decision (Cohen et al., 2002; Dehaene et al., 2002), or structure-based word decision (Binder et al., 2006; Kronbichler et al., 2008). This region has also been called visual word form area or VWFA (Cohen et al., 2000).

It has been proposed that the activation patterns of these three regions of the reading system are different between good and deficient readers, such as dyslexics (Fiez and Petersen, 1998; Shaiwitz et al., 1998). Deficient readers present fewer activations of occipital-temporal and temporal-parietal regions (Shaiwitz et al., 2002) and more activations of the inferior frontal regions (Temple et al., 2001). This pattern has been interpreted as a compensatory mechanism (Shaiwitz et al., 2006), although other authors have also proposed the existence of bilateral activations in these three regions with the same purpose (Gebauer et al., 2012).

Fu et al. (2006) found statistically significant estimates when comparing the visual word recognition task’s difficulty and pointed out that areas such as the precuneus, the anterior cingulate cortex, and the left middle frontal gyrus are functionally connected to one another. Additionally they pointed out that areas such as the left inferior frontal gyrus, the left middle temporal gyrus, and the right middle areas also present statistically significant connections according to task difficulty. In a paper on reading competences, Levy et al. (2009) showed functional connectivity models which included areas such as the left middle occipital gyrus, the left occipital-temporal junction, the left parietal cortex, and the left inferior frontal gyrus. Dick et al. (2010) – by using structural equation models (SEMs) – found statistically significant connectivity patterns involving the inferior frontal gyrus (pars triangularis/pars opercularis/ventral premotor), the supramarginal gyrus, the posterior superior temporal, the anterior superior temporal, the posterior superior temporal sulcus, the posterior middle temporal gyrus, the fusiform gyrus, and the visual, striate, and extrastriate cortex. In adition Vitali et al. (2010) – in a two-case study – yields a regular connectivity structure focused basically on significant effects between areas such as the inferior frontal gyrus, the middle temporal gyrus, and the inferior parietal lobule. Taken together, these papers suggest some regularity in the areas involved in this type of tasks (Taylor et al., 2010).

However, the vast majority of the previously mentioned studies have been conducted on shallow orthographies. Some authors argue that the consistency of different orthographies is a factor that may directly influence the processing of reading (Davies et al., 2007). Orthographic consistency and its corresponding instructional regime could lead to the adoption of different reading strategies across languages based on visual or whole word recognition in shallow orthographies, and on phonological recognition in transparent ones (Wimmer and Goswami, 1994). These concepts are consistent with different reading models, such as the DRC (Coltheart et al., 2001), on which anatomical (Allen et al., 2009) and functional (Fiebach et al., 2002) representations were studied.

Spanish is considered a language with a regular orthography, such as Dutch, Italian, and German, due to its high grapheme–phoneme correspondence for reading; however, for writing, some phonemes may be mapped onto two or three different letters. This is particularly true in Mexican Spanish, given that – in addition to the matches between a phoneme and several graphemes of standard Spanish – other sounds are also equivalent. For example, the phoneme /s/ matches the graphemes “c,” “s,” and “z”; the phoneme /x/ matches “x,” “g,” and “j” the phoneme /j/ matches “y” and “ll”; and the phoneme /b/ matches “b” and “v.” Moreover, Mexican Spanish comprises a great percentage of words originating from the country’s various indigenous languages – now completely integrated into Spanish – many of which involve these types of phonemes and spelling not based on orthographic rules (arbitrary). Because of all this, Mexican Spanish is probably the transparent language where speakers might make the most mistakes when writing pseudohomophones (words with an orthographic error and the same phonology as the correct one), or in the visual recognition of a pseudohomophone as a valid word while reading.

Although these mistakes do not compromise reading comprehension in normal persons in a meaningful way, they do cause the speakers of Mexican Spanish to make numerous pseudohomophone spelling mistakes, something observable in the general population (Gómez-Velázquez et al., 2013a).

The study by González-Garrido et al. (2014) suggests that the electrophysiological correlates of orthographic error processing have shown that adults with low orthographic abilities have problems in detecting orthographic rules violations, which could indicate weak representations in the orthographic lexicon, or a difficulty in automatically accessing such representations. According to Bahr et al. (2012), the proper development of an orthographic lexicon could lead to an adequate processing of phonology, orthography and morphology at the level of visual word recognition. In addition, few works exist on effective connectivity in visual word recognition tasks using SEM or Dynamic Causal Model (DCM). The paper by Kiebel et al. (2007) yields statistically significant connections in this type of task in areas like the left primary auditory cortex, the right primary auditory cortex, the left superior temporal gyrus, the right superior temporal gyrus, and the right inferior frontal gyrus. An innovative aspect of this paper is that, in order to estimate those effects, the authors used DCM estimation techniques based on assumptions somewhat different from those of SEM.

The main objective of the present study was to explore the possible differences in effective connectivity among subjects with high and low spelling orthographic abilities while processing pseudohomophone orthographic errors by using visual word recognition tasks. Another goal of this study is to compare SEM and DCM models given that they entail different approaches to connectivity models and each one reports on different processes to model the brain activities under the Bold signal paradigm.

## Materials and Methods

### Participants

Twenty-four young adults (age *M* = 21.83 years, SD = 5.02, 10 women) participated in the experiment. They were all right-handed in agreement with the Edinburgh handedness inventory (Oldfield, 1971), with normal or corrected view, and none presented a history of neurological illness or learning disorder. They all signed an informed consent and received economic compensation for their participation, in accordance with the permission and recommendations of the Ethical Committee of the Instituto de Neurociencias of the University of Guadalajara, Mexico.

Prior to the fMRI registrations, five tasks were applied to all the participants intended to assess their handling of homophone spelling in Spanish words (b-v, c-s-z, g-j, ll-y, h-no h) in four different contexts: completing words, dictation (words and text), error detection in a text, and free composition. The tasks used to discriminate the subjects’ performances had yielded an adequate reliability value (α = 0.833) and a very high discrimination capacity in order to distinguish between groups with different orthographic skills (*t* = 11.608; *p* < 0.001) in a previous study, complementary to the current one, with a general sample of 827 subjects (Gómez-Velázquez et al., 2013b).

Out of this large sample, 12 subjects with low spelling skills (LSSs group, 10th percentile) and another 12 with high spelling skills (HSSs group, 90th percentile) were selected to form this study’s sample.

### Stimuli and Procedure

Throughout two experimental tasks, the subjects were exposed to 80 Spanish words, 60 out of which were spelled correctly. Also, 20 words contained a homophone orthographic error [e.g., *sapato* (incorrect) instead of *zapato* (correct), the Spanish word for ‘shoe’].

In the first task (blocks A and B, spelling recognition task), the participants were required to indicate whether the word was written correctly or else contained a pseudohomophone orthographic error. In block A, 50% of the words were written correctly and the remaining 50% contained an orthographic error. In block B, 100% of the words were written correctly. In the second task (blocks C and D, visuoperceptual recognition task) the participants were instructed to answer whether the word displayed contained the vowel “i” or not. In block C, 50% of the words were written correctly and 50% contained a pseudohomophone orthographic error. In block D, 100% of the words were written correctly. We should bear in mind, however, that the participants did not have that information, neither in block B nor D.

Both the stimuli and the interval between them were 1 s long. In order to present them, a block design was used: the stimuli were divided into eight blocks of 10 stimuli each and presented pseudo-randomly. The stimuli were presented in an Arial 60 font and were typed in white on a black background with a 300 pixel-per-inch resolution.

Both the words spelled correctly and those with an orthographic error had a high or low frequency according to a frequency dictionary widely used in studies involving words in Spanish (Sebastián et al., 2000).

The total number of stimuli from both categories (words spelled correctly and incorrectly) was divided in half to be presented in both experimental tasks. In each task, four rest blocks were presented with a center fixation dot during which the subjects were not supposed to conduct any activity; the change of color in the fixation dot told the subjects they were about to start watching words and executing answers. Likewise, four activation blocks were presented in each task with ten stimuli each: two of them with words spelled correctly and incorrectly (50–50%), and two blocks only with words spelled correctly.

**FIGURE 1:**
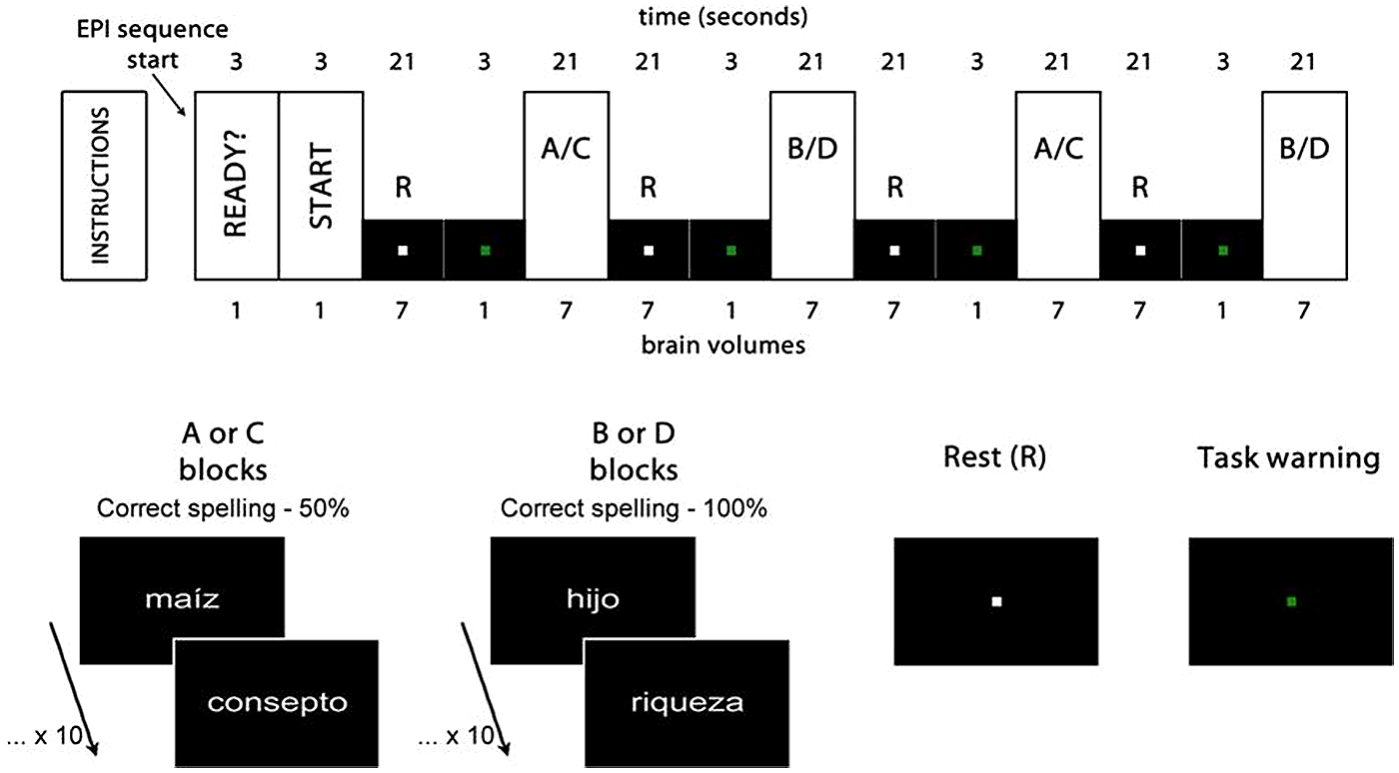
**Experimental design.** Rest (R) and activation (A, B, C, and D) blocks. The first two brain volumes were eliminated from the analysis, as well as the four task warning volumes. *Maíz* (corn), *hijo* (son) and *riqueza* (wealth) are examples of words spelled correctly. *Consepto* (concept) is an example of a word spelled incorrectly, with an *s* instead of a *c*, thus generating a homophone error.

The participants gave their answer to each stimulus through one out of two buttons, following the requirement of the two experimental tasks: *spelling recognition*, where they had to decide, as quickly as possible, whether the word presented was spelled correctly or incorrectly (**Figure 1**, A and B blocks); and *visuoperceptual recognition*, where they had to decide, also quickly, whether the words presented contained the vowel ‘i’ or not (**Figure 1**, C and D blocks). The complete design of both tasks can be observed in **Figure 1**.

### Image Acquisition Through Magnetic Resonance Imaging

We used a GE Signa Excite HDxT 1.5 Tesla (GE Medical Systems, Milwaukee, WI, USA) and an 8-channel head coil. For each experimental task, we obtained 32 4-mm thick adjacent axial cuts. We used an echo planar pulse sequence with a Repetition Time of 3 s, echo time of 60 ms, 26-cm FOV, and a 64 × 64 matrix. The voxel size used was 4.06 × 4.06 × 4 mm. From each experimental task, a total of 62 brain volumes were obtained. For reasons of image acquisition time and experimental design, six brain volumes per task were discarded, thus leaving a total of 56 to be analyzed later on (according **Figure 1**).

The pre-process and the statistical analysis of the images were conducted using the SPM8 computer package (http://www.fil.ion.ucl.ac.uk/spm/software/spm8/). The images were realigned spatially, readjusted to the voxel size, and normalized according to the MNI reference – *Montreal Neurological Institute* – and Talairach coordinates.

For the smoothing, a Kernel *Gaussian* filter three times the voxel size was used on the *x, y*, and *z* axes. Based on the analysis of each group in each task, regions of interest (ROIs) were formed by means of the MarsBar software (Brett et al., 2002), and therefore the selection of ROIs was data driven. All the models – SEM and DCM – for the study of effective connectivity were data driven. To conduct this analysis, we previously carried out four first-level analyses (Zarabozo-Hurtado et al., under revision), differentiating groups and tasks, in fact one first level analysis was done for HSS group and spelling recognition task comparing the activation between the blocks A and B, the same analysis was done for the LSS group, and the same structure was repeated for the visuoperceptual recognition task (first level HSS group comparing C and D Blocks and first level LSS group comparing C and D Blocks).

### Structural Equation Models and Dynamic Causal Models Approach

There are several statistical conditions involved in the use of SEM to estimate connectivity. Basically, they concern the SEM properties as regards the linear model, and the conditions of range and order that the Path Analysis models must follow. In fact, generating a factorial structure to obtain a score by ROI based on the voxels defined would be, ultimately, a peculiar application of the dimension reduction or, in SEM terms, of a measurement model. Accordingly, it is more than debatable that effective connectivity estimation follows a Path Analysis model strictly.

Regardless of these rather conceptual considerations, several papers have shown the limitations of this technique, which can be summarized in the following aspects. Firstly, SEMs do not allow us to easily analyze the self-impact effects, that is, the β*_ii_* effects where a specific ROI shows an effect on itself. This type of effect does not comply with the condition of order and compromises the estimation. Secondly, we should remember that non-recursive models are difficult to estimate and, in the case at hand, they are inevitable effects for a reasonable approach to functional connectivity. This type of condition has been formulated differently in papers on connectivity (Horwitz et al., 1999; Gonçalves and Hall, 2003; Astolfi et al., 2004, among many others). Additionally, some statistical questions arise, in the sense that the ROI selection usually conducted in SEM may generate some bias in the parameter estimation.

Consequently, papers like those by Erickson et al. (2005), Caclin and Fonlupt (2006), or Marrelec et al. (2006, 2007) show us how the estimation of correlation between ROI values is not bias-free depending on the number of brain volumes involved in the analysis and on the number of ROIs selected. Accordingly, the estimations of partial correlations or the results with estimation techniques that reduce collinearity effects are not necessarily similar to those obtained with maximum likelihood (ML) estimations.

In addition, an effect exists that has received little attention based on the assumption of variance homogeneity between ROIs which can only be solved by standardizing the values, but which is not assumable from the onset (Charlton et al., 2008). Even the definition of ROI itself is only a somewhat paradoxical effect which is not necessarily constant through the subjects. Accordingly, the selection of a number of ROIs within a group of subjects involves an essential variability of significance and activations (Marco et al., 2009). That is to say, ROIs with significant effects in one subject do not necessarily present activations in another or with the same intensity if the activation is repeated.

Such is the case between groups (Kim and Horwitz, 2009) and between experimental conditions, which entails a difficult situation to manage in statistical terms. Despite these comments, effective connectivity models based on SEMs have proven useful for their verification, and many are the papers which can be considered as good praxis from a statistical point of view (Rowe, 2010; Carballedo et al., 2011; Karunanayaka et al., 2011; Inman et al., 2012), in addition to the modifications and extensions generated (Chen et al., 2011), or the use of Extended Unified SEMs (Gates et al., 2011).

The choice of DCM models seems an interesting alternative to the SEM models, given that their statistical properties make them somewhat more malleable. Generally speaking, they are more tolerant with reciprocal effects, with *β_ii_* effects, and they incorporate the direct effects from the stimuli in the activations of specific ROIs. Additionally, the incorporation of the B matrix into the general model allows us to establish the mediating effects of the experimental conditions on the direct effects between ROIs. Indeed, the DCM-derived general model can be summarized as follows:

(1) $$Z_{t}\,\;=\;(A\,\;+\;\Sigma u_{t(j)}B^{j})Z_{t}\,\;+\;Cu_{r}$$

where t is continuous time, *Z_t_* is the neuronal activity, *u_t(j)_* is the j input at time t, *A,B^j^*, and *C* are the connectivity matrices, *A =* Intrinsic connections, *B =*Modulatory Connections, and *C =*External Connections.

Therefore, as mentioned above, not specifying the C matrix involves that, given certain conditions – that is, *y_i_ = z_i_* and *ζ = Cu_t_* –, the expression [1] becomes a general SEM expression, i.e., the popular *y_t_ = βy_t_ + ζ_t_*, in LISREL terminology. Consequently, the advantage of using DCM over SEM is based on the idea of defining the impact of External Connections within the model, which in SEM complicates estimation.

On the other hand, DCM is strictly linked to ROIs presenting statistically significant activations. In general, the fundamental condition of applying DCM lies on the situation of subject-model specificity when fitting models to specific subjects under defined experimental conditions.

This question has not been overlooked. In fact, several choices of parameter estimation have been generated for it. Penny et al. (2009), Marreiros et al. (2010), or Friston et al. (2012) are examples of possible DCM approaches to different scenarios of complexity.

It has also been discussed that using DCM entails difficulty given that it only analyzes activated ROIs, and therefore, it overlooks other sources of variation. However, some proposals have tried to generate alternatives to this possible bias effect (Wolff et al., 2010), including the inevitable Bayesian estimations effect (Patel et al., 2006). These should be taken into account to a greater extent than they are currently, since they would solve certain matters concerning the classic statistical significance used nowadays or the study of DCM in seeded or unseeded resting situations for a wider study of ROIs.

According to the recommendations by Stephan et al. (2010), we are interested in inferring neurophysiological mechanisms in terms of the statistical inference effect (excitatory or inhibitory impact) using the information derived from the SEM models as a prior. We are focusing on determining the task’s impact causal effect, with group distinction according to the effective connectivity between statistically significant ROIs in a first-level analysis of the comparison of blocks in each task. This analysis is conducted separately in each group.

Despite the limited empirical evidence in this field (using orthographic tasks in Spanish-speaking populations) we expected the DCM models to be different for the two groups considered, that is, more complex in the A–B task for the LSS group than for the HSS group. In contrast, for the C–D task, we expected the models fitted in the HSS group to be more complex than those in the LSS group because the former had to complete both tasks (spelling and visuoperceptual recognition).

In any case, Saur et al. (2010) – in an auditory comprehension task – find a statistically significant effect in several ROIs, which is very similar to what our data and results show. Despite that, our objective has no strict anatomical support. As is usual in many other connectivity models, little evidence exists about the anatomic viability of the DCM results.

## Results

### Behavioral

A multivariate analysis of the variance (MANOVA) was conducted by using orthographic competence (High or Low) as a factor between the two groups and the four programmed blocks as an intra-group factor (A, B, C, and D), defining the subjects’ ages as a covariant to extract the components caused by that factor and the following dependent variables: the number of correct answers given in each block, and the simple reaction time in each subject’s answer to the 20 tries in each experimental condition.

Clearly significant was the interaction between Group and Blocks concerning the number of correct answers (*F* = 5.017; *p* = 0.010; η^2^ = 0.442). The HSS group presents better performance in both spelling recognition tasks blocks than the LSS group, whereas performance is similar for both groups in the visuoperceptual recognition task. Also for the reaction time, the interaction between the Group and the Blocks was statistically significant (*F* = 25.554; *p* < 0.001; η^2^ = 0.801).

Finally, the main effect linked to the group effect for the reaction time (*F* = 13.367; *p* = 0.001; η^2^ = 0.389) was also statistically significant. The HSS group was faster than the LSS group in both blocks of the spelling recognition tasks but, in the visuoperceptual recognition task, the HSS group was slower in block C than the LSS group, and the opposite effect in block D.

Neither the effect of age as a covariant nor its interaction with the block or the group of belonging turned out statistically significant. To prevent the possible “double dipping” effect described by Kriegeskorte et al. (2009), all contrasts were made by orthogonal coefficients so that the effects were not overestimated and, likewise, the significances of this phase were carried out under the false discovery rate (FDR) criteria with *p* < 0.001. A more detailed display of these results may be consulted in a previous analysis in (Zarabozo-Hurtado et al., under revision). **Table 1** presents a brief statistical description (mean and SD) of the behavioral results for an analysis of the aforementioned performance of the different groups.

**Table 1:** Descriptive statistical results, mean and standard deviation (SD) for the number of correct answers and the reaction time for each experimental condition.

Group	Number of correct answers				Average reaction times				AGE
	A	B	C	D	A	B	C	D	
High spelling skills (HSS)	17.33 (0.98)	15.75 (4.18)	17.00 (2.13)	18.00 (2.29)	825.83 (63.86)	780.98 (63.74)	688.64 (54.67)	649.33 (46.28)	22.50 (1.61)
Low spelling skills (LSS)	6.76 (2.89)	10.67 (3.37)	18.67 (1.43)	18.17 (1.03)	847.91 (43.22)	809.99 (40.22)	664.24 (42.20)	682.77 (53.37)	21.17 (1.31)


**FIGURE 2:**
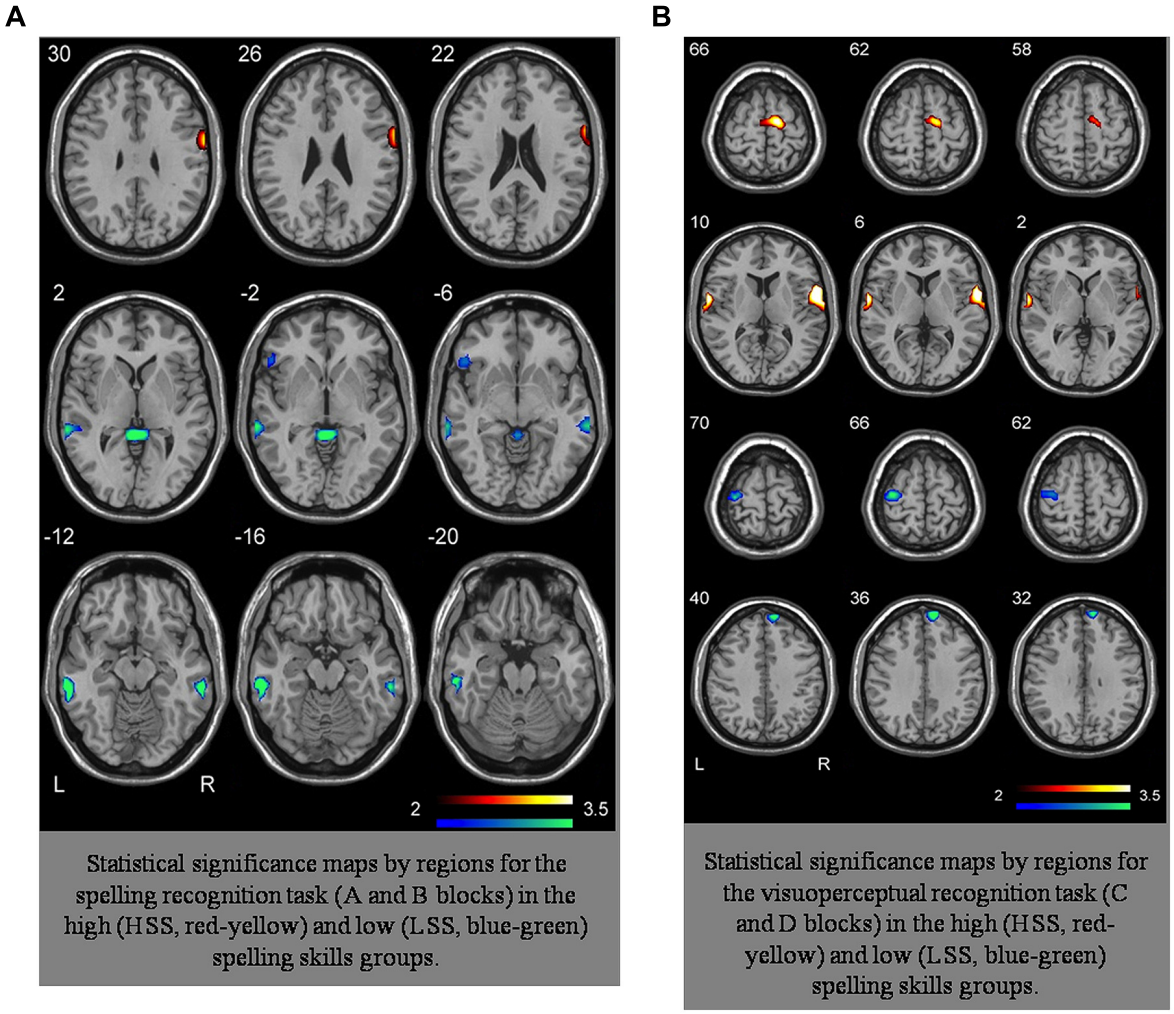
**(A)** Representation of activation loci in A–B tasks HSS group and LSS group. **(B)** Representation of activation loci in C–D tasks HSS group and LSS group.

**FIGURE 3:**
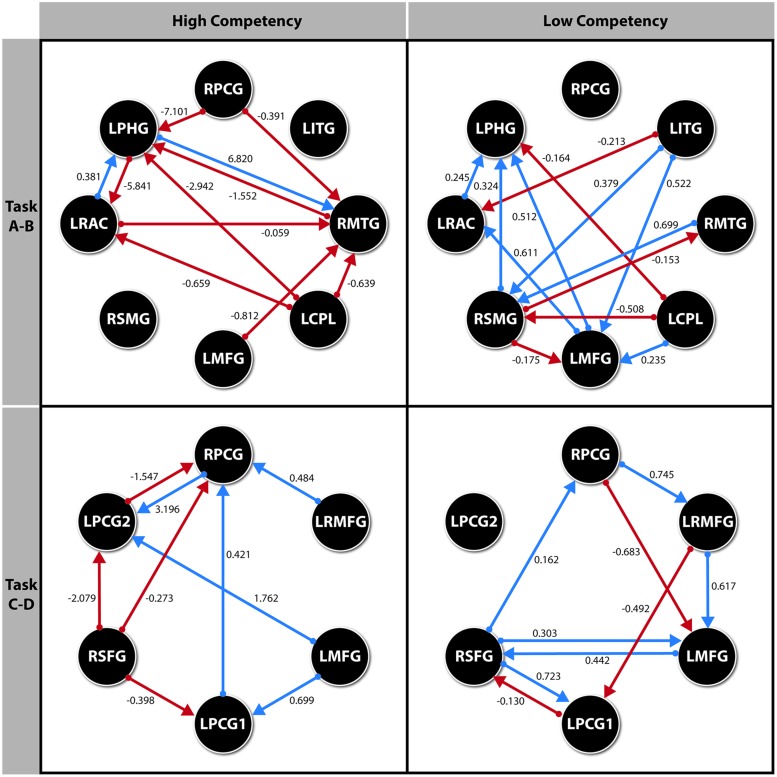
**Path diagram for each model with the parameter values for each significant direct effect.** All effects *p* < 0.05. ROI’s for Spelling recognition Task. RPCG (Right Precentral Gyrus), LITG (Left Inferior Temporal Gyrus), RMTG (Right Middle Temporal Gyrus), LCPL (Left Cerebellum Posterior Lobule), LMFG (Left Middle Frontal Gyurs), RSMG (Right Supramarginal Gyrus), LRAC (Left–Right Anterior Cingulate), LPHG (Left Parahippocampal Gyrus). ROI’s for Visuoperceptual recognition Task. RPCG (Right Precentral Gyrus), LRMFG (Left–Right Middle Frontal Gyrus), LMFG (Left Middle Frontal Gyrus), LPCG1 (Left Precentral Gyrus 1), RSFG (Right Superior Frontal Gyrus), LPCG2 (Left Precentral Gyrus 2).

We conducted an analysis of the SPM algorithm’s linear model (Friston et al., 2007). All the analyses were conducted for each group and task comparing the two blocks in each task. For each group we worked with the average image of each subject obtained in the first-level analysis. The first-level analysis through multiple comparisons with complete factorial ANOVA differentiated by groups and tasks – with a final FDR of α = 0.001 – showed that the statistically significant activations appeared bilaterally, mainly in two large groupings located in the inferior temporal gyrus, predominantly in the left hemisphere, and in the middle temporal gyrus, predominantly in the right hemisphere. In the LSS group, we also spotted activations in the right hemisphere’s supramarginal gyrus and in the middle portion of the frontal gyrus in the left hemisphere. Likewise, this group presented activations in subcortical regions such as the cerebellum, the parahippocampal gyrus, and the anterior cingulate region, all of them in the left hemisphere. Conversely, the group analysis of HSS unveils activation in a small grouping located in the right hemisphere’s pre-central gyrus. The exact location of these activations can be seen in **Table 2** (Zarabozo-Hurtado et al., under revision) and are represented in **Figures 2A,B** and **3**.

**Table 2:** Definition of Regions of interest (ROI) from activations by group and task.

ROI number	*Task* Hemisphere/Anatomical region (ABREV.)/*t* values	MNI coordinates ranges		
		*x*	*y*	*z*
**Spelling recognition -A and B blocks-**				
1	R/Precentral gyrus (RPCG; *t* = 2.89)	64/68	-6/10	10/30
2	L/Inferior temporal gyrus (LITG; *t* = 4.56)	64/-50	-32/-50	0/-18
3	R/Middle temporal gyrus (RMTG; *t* = 4.04)	52/70	15/-41	-2/-22
4	L/Cerebellum, posterior lobule (LCPL; *t* = 3.24)	-42/-22	-46/-36	-38/-30
5	L/Middle frontal gyrus (LMFG; *t* = 3.34)	-50/-22	-10/10	42/58
6	R/Supramarginal gyrus (RSMG; *t* = 3.07)	48/62	-60/-44	26/36
7	L–R/Anterior cingulate (LRAC; *t* = 2.92)	-4/6	30/38	-10/14
8	L/Parahippocampal gyrus (LPHG; *t* = 2.89)	-24/-14	-18/6	-22/-14
**Visuoperceptual recognition -C and D blocks-**				
1	R/Precentral gyurs (RPCG; *t* = 4.23)	52/86	-18/10	2/26
2	L–R/Middle frontal gyrus (LRMFG; *t* = 3.48)	-16/24	-26/2	46/74
3	L/Middle frontal gyrus (LMFG; *t* = 3.39)	-50/-34	6/18	30/54
4	L/Precentral gyrus [1] (LPCG1; *t* = 3.30)	-66/-58	-18/2	-6/14
5	R/Superior frontal gyrus (RSFG; *t* = 3.74)	4/16	50/56	22/34
6	L/Precentral gyrus [2] (LPCG2; *t* = 3.53)	-46/-26	-14/-26	52/70	

ROI box measures are given in pairs of MNI coordinates for *x, y*, and *z* vectors (e.g., *x*: 64/68, from 64 to 68). Each MNI coordinate represents voxel ranges that define a significant cluster. All *t* values *p* < 0.001.

The extraction of ROIs was data driven. We selected the most significant signal values for each region by defining an area of 5 mm and conducting a Component Principal Analysis to extract the ROI. For each cluster we presented the *t* value for each group, first the HSS group and then the LSS group. In all the cases, we obtained *p* < 0.00001 after Bonferroni correction. The minimum number of voxels per significant cluster was 22.

Based on the results above and consequently following a data-driven strategy, we extracted the ROIs values for the four first-level analyses conducted through MarsBar by defining, for each significant area, a 5-mm volume around the most statistically significant *voxel* for each significant cluster. We used a threshold = 3, and obtained the value derived from the first component of the principal component analysis (PCA). Thus we generated the resulting score in a standardized scale with a mean = 100. In all the cases, the percentage of variance explained by the first component ranged between 84.5 and 92.1%, which guaranteed the first component’s reliability. Likewise, we estimated, for each task and group, Pearson’s product-moment correlations matrix in order to begin the parameter estimation. **Table 3** displays the values of the correlations estimated between the previously defined ROIs.

**Table 3:** Correlation matrix between ROIs for the AB task (spelling recognition) and for the CD task (visuoperceptual recognition) for the two competence groups (High or Low).

Correlation between ROIs for the Spelling recognition Task – A and B blocks –(HSS group – LSS group)								

	**RPCG**	**LITG**	**RMTG**	**LCPL**	**LMFG**	**RSMG**	**LRAC**	**LPHG**
RPCG	1							
LITG	0.672^∗^*0.137^∗∗^*	1						
RMTG	0.722^∗^*0.411^∗^*	0.325^∗∗^*0.027*	1					
LCPL	0.533^∗^-*0.189^∗∗^*	0.355^∗∗^*0.226^∗∗^*	0.230^∗∗^*0.105^∗∗^*	1				
LMFG	-0.737^∗^-*0.014*	0.148^∗∗^*0.529^∗^*	-0.597^∗^-*0.060*	-0.501^∗^*0.417^∗^*	1			
RSMG	0.666^∗^*0.232^∗∗^*	0.174^∗∗^*0.283^∗∗^*	0.566^∗^*0.612^∗^*	-0.012-*0.348^∗∗^*	-0.449^∗^-*0.110^∗∗^*	1		
LRAC	-0.545^∗^*0.147^∗∗^*	-0.122^∗∗^*0.109^∗∗^*	-0.046-*0.047*	-0.812^∗^*0.209^∗∗^*	0.404^∗^*0.496^∗^*	0.066-*0.108^∗∗^*	1	
LPHG	-0.533^∗^-*0.178^∗∗^*	-0.116^∗∗^*0.359^∗∗^*	-308^∗∗^*0.151^∗∗^*	-0.402^∗^-*0.014*	0.240^∗∗^*0.525^∗^*	-0.266^∗∗^*0.298^∗∗^*	0.498^∗^*0.426^∗^*	1

**Correlation between ROIs for the Visuoperceptual recognition Task – C and D blocks –(HSS group – LSS group)**								

	**RPCG**	**LRMFG**	**LMFG**	**LPCG1**	**RSFG**	**LPCG2**		

RPCG	1							
LRMFG	0.162*0.772^∗^*	1						
LMFG	-0.215-*0.127*	-0.327^∗∗^*0.223*	1					
LPCG1	-0.183-*0.312^∗∗^*	-0.251-*0.346^∗^*	0.500^∗^*0.313^∗∗^*	1				
RSFG	0.294^∗∗^*0.081*	0.001*0.220*	0.412^∗^*0.637^∗^*	-0.131*0.585^∗^*	1			
LPCG2	-0.509^∗^-*0.038*	0.146*0.235*	0.095*0.350^∗^*	0.330^∗∗^*0.320^∗∗^*	-0.365^∗^*0.184*	1		


*^∗^p < 0.01, ^∗∗^p < 0.05*.

#### ROIs for the Spelling Recognition Task

RPCG (Right Precentral Gyrus), LITG (Left Inferior Temporal Gyrus), RMTG (Right Middle Temporal Gyrus), LCPL (Left Cerebellum Posterior Lobule), LMFG (Left Middle Frontal Gyurs), RSMG (Right Supramarginal Gyrus), LRAC (Left–Right Anterior Cingulate), LPHG (Left Parahippocampal Gyrus).

#### ROIs for the Visuoperceptual Recognition Task

RPCG (Right Precentral Gyrus), LRMFG (Left–Right Middle Frontal Gyrus), LMFG (Left Middle Frontal Gyrus), LPCG1 (Left Precentral Gyrus 1), RSFG (Right Superior Frontal Gyrus), LPCG2 (Left Precentral Gyrus 2).

### Structural Equation Model Estimation

In order to estimate effective connectivity, each correlation matrix was submitted to the procedure described by Inman et al. (2012) in order to obtain the best possible model regarding fit. To achieve that, we opted for a conventional ML estimation based on the previous correlations; we set unrestricted parameter estimation and adopted the usual assumptions of SEM. In this case, they had been adapted to the Path Analysis’ characteristics, given the lack of latent variables. Therefore, we assumed that *E(Y_i_) = 0* and *Var(Y_i_) = 1*. In consequence, all the variables were reduced and normalized and *E(ζ_i_ζ_j_) = E(Y_i_ζ_j_) = 0*; the structural errors are not correlated between themselves or between the observed variables. For these calculations, we used the Mplus software bootstrap estimation and simulation routines for the standard errors, and we analyzed each possible effect combination with regard to the null model. Additionally we also analyzed, in each case, the values of the indexes of fit and the results of Akaike’s criteria (AIC), and the Bayesian information criteria (BIC).

The results of the models with the best fit are summarized in **Table 4**. To select them, we eliminated those models which turned out unidentified, those which did not converge in the estimation process, and those whose solution presented incorrect fits. Specifically, we discarded those whose *p* value associated to the *χ^2^* fit test was below 0.10. Additionally, we also discarded those whose values in the Tucker Lewis index (TLI), comparative fit index (CFI), goodness of fit index (GFI), and adjusted goodness of fit index (AGFI) which were not over.95. Lastly, we also discarded those whose standardized mean residuals (SMRs) values were not below 0.10.

**Table 4:** Fit index for the best models under SEM approach for each task and groups.

	Spelling recognition Task – A and B blocks –											
Group	Fit index				Explained variance (*R^2^*)							
	χ^2^	*df*	*p*	RMSEA	RPCG	LITG	RMTG	LCPL	LMFG	RSMG	LRAC	LPHG
HSS	0.786	1	0.3754	0.0–0.09			0.543				0.668	0.436
LSS	6.362	7	0.4982	0.0–0.04			0.399		0.402	0.681	0.280	0.464
	
	**Visuoperceptual recognition Task – C and D blocks –**											
	
**Group**	**Fit index**				**Explained variance (*R^**2**^*)**							
	**χ^**2**^**	***df***	***p***	**RMSEA**	**RPCG**	**LRMFG**	**LMFG**	**LPCG1**	**RSFG**	**LPCG2**		

HSS	4.140	3	0.2468	0.0–0.07	0.474			0.429		0.521		
LSS	0.950	1	0.3298	0.0–0.09	0.409	0.589	0.462	0.520	0.225			


Out of all the models complying with the above criteria, we selected, for each condition, those offering the best fit and the highest value in the determination coefficient (*R^2^*) estimated for each endogenous variable.

#### ROIs for the Spelling Recognition Task

RPCG (Right Precentral Gyrus), LITG (Left Inferior Temporal Gyrus), RMTG (Right Middle Temporal Gyrus), LCPL (Left Cerebellum Posterior Lobule), LMFG (Left Middle Frontal Gyurs), RSMG (Right Supramarginal Gyrus), LRAC (Left–Right Anterior Cingulate), LPHG (Left Parahippocampal Gyrus).

#### ROIs for the Visuoperceptual Recognition Task

RPCG (Right Precentral Gyrus), LRMFG (Left–Right Middle Frontal Gyrus), LMFG (Left Middle Frontal Gyrus), LPCG1 (Left Precentral Gyrus 1), RSFG (Right Superior Frontal Gyrus), LPCG2 (Left Precentral Gyrus 2).

Finally, the path diagrams for each model representing the standardized parameters ML estimation are shown in the **Figure 3**.

As we can observe in **Figure 2**, the complexity of the connectivity network is more complex in the LSS group than in the HSS group for the spelling recognition task (AB blocks). In the LSS, 13 paths (seven with positive effect) were defined among the eight ROIs analyzed but in fact the RPCG ROI is not connected to the rest of ROIs. 11 paths (three of them with positive effect) were defined in the HSS group among the ROIs, but in fact the RSMG ROI is not connected to the rest of ROIs.

As for the HSS group, only three ROIs received connections from the other ROIs (right parietal gyrus, LRAC, and RMTG), whereas in the LSS group, five ROIs received connections from the other ROIs involved in the system (right parietal gyrus, LRAC, RMTG, LMFG and RSMG).

For the visuoperceptual recognition task the complexity of the connectivity network is similar in both groups. Eleven paths were defined for both, but the connectivity structure is different: in the LSS group, all the ROIs involved in the connectivity network receive connections from at least one of the other ROIs of the system.

### Dynamic Causal Modeling

This paper analyzes the DCM models in each of the two groups of orthographic competence (High and Low) in the two tasks presented: A–B (spelling recognition task), and C–D (visuoperceptual recognition task). Therefore, four DCM models were generated based on the average values of each group’s twelve subjects under every condition.

However, the analysis of the results obtained by SEM indicate that, in the case of the subjects with high orthographic competence when solving the A–B task, their levels of significance are of little statistical intensity, except for the ROI defined by the right pre-central gyrus (Zarabozo-Hurtado et al., under revision).

Thus being the case, and in light of the presence of only one statistically relevant ROI, we decided to discard the DCM model for this group and task, so that, ultimately, the remaining three models were studied with several significant ROIs. To do this, we followed the recommended steps by Stephan et al. (2010) in order to guarantee the definition of space and the causal effects in the parameter structure inference, using an optimal Bayesian parameter averaging and defining fixed effects, as usual.

**FIGURE 4:**
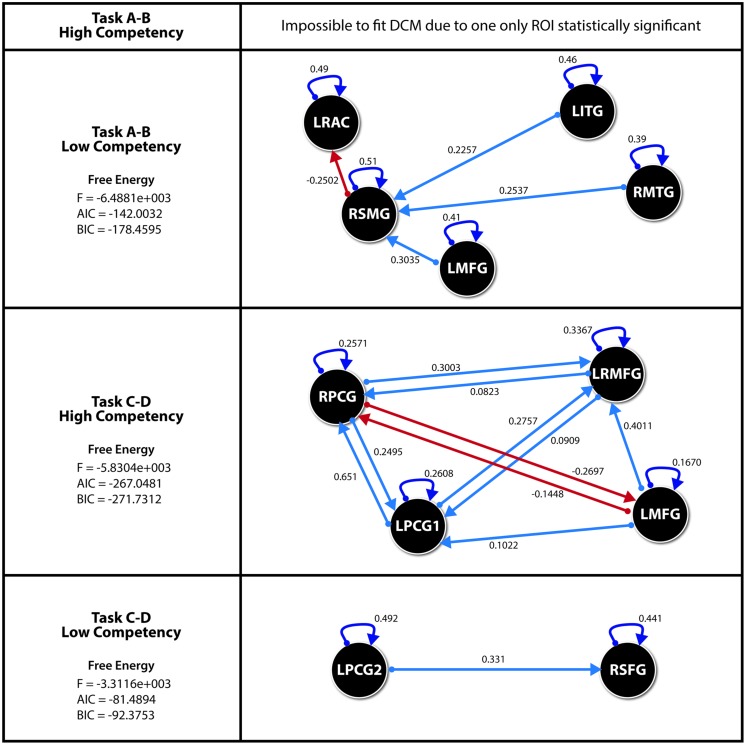
**Effects for each DCM with the parameter values for each significant direct effect.** All effects *p* < 0.05. ROI’s for Spelling recognition Task. LITG (Left Inferior Temporal Gyrus), RMTG (Right Middle Temporal Gyrus), LMFG (Left Middle Frontal Gyurs), RSMG (Right Supramarginal Gyrus), LRAC (Left–Right Anterior Cingulate). ROI’s for Visuoperceptual recognition Task. RPCG (Right Precentral Gyrus), LRMFG (Left–Right Middle Frontal Gyrus), LMFG (Left Middle Frontal Gyrus), LPCG1 (Left Precentral Gyrus 1), RSFG (Right Superior Frontal Gyrus), LPCG2 (Left Precentral Gyrus 2). Akaike information criteria (AIC), Bayesian information criteria (BIC).

The estimations of the three remaining DCM models are displayed in **Figure 4**. As we can see the connectivity network for the visuoperceptual recognition task is more complex in the HSS group than in the LSS group. In the HSS group, four ROIs are involved in the system and interconnected to each other. Instead, for the LSS group, only two interconnected ROIs are involved in the system.

It is also important to mention than the ROIs involved are different in both groups.

## Discussion and Conclusion

We studied the possible differences in detecting homophone orthographic errors in the neurobiological substrate by using two approaches: analyzing the effective connectivity model estimated through SEM and DCM. In order to study this phenomenon, two tasks were used: a spelling recognition task, and a visuoperceptual recognition task. These were applied to two groups of subjects, one with HSS, and another with LSS (Zarabozo-Hurtado et al., under revision).

According to the results while performing the spelling recognition task, the LSS group showed poorer behavioral performance (fewer correct answers and higher reaction times) as compared to the HSS group. Given that the early stages of the reading process involve encoding and orthographical-phonological conversion, these data suggest that, globally, this cognitive process is different in the LSS and HSS groups.

The SEM results of the HSS group suggest that, in the spelling recognition task, two brain areas are involved in the majority of significant effects: the RMTG, and, to a lesser extent, the LPHG. In the LSS group, however, the majority of significant effects are received in the LPHG, and, to a lesser extent, the RSMG. The latter anatomical region has been reported in different studies under transparent orthographies as visual to a phonological encoder (Wimmer et al., 2010; Gebauer et al., 2012).

In this sense, we should mention that the LSS group probably presents activations in the supramarginal gyrus as a compensatory mechanism, since these subjects do not present the temporal-occipital activations shown by the HSS group and which are usually observed in reading tasks in healthy persons. For us this means that the LSS subjects must use this compensatory mechanism to access other later reading processes. This is a consistent result in papers studying reading in transparent languages and, in this sense, our results are similar to those in Booth et al. (2002, 2004) and Gebauer et al. (2012).

We should point out that such compensatory mechanism in readers of transparent orthographies is observed rather generally in persons with reading deficiencies, as shown by the recent meta-analyses by Maisog et al. (2008) and Richlan et al. (2009).

Additionally, the intensity of these effects both in the most active brain areas during the task and in the rest of ROIs is much less intense in the LSS group than in the HSS group. In the spelling recognition task, the connectivity model is very different between the HSS and the LSS groups. On the one hand, in the former, the ROI receiving the most significant effects is the LPHG, whereas for the LSS group, it is the LMFG. Once again, the most intense effects appear in the HSS group, while they are much more diffuse in the LSS group.

Along with the behavioral data, and given that the early stages of the reading process involve encoding and orthographical-phonological conversion, these data suggest that, globally, we can conclude that the effective connectivity pattern during the spelling recognition task is different between HSS and LSS subjects. The behavioral performance data, which shows that the LSS group performed worse in the task than the HSS group, all of it suggests that the reading process is globally different between the HSS and LSS groups. This had already been proposed in previous neuroimaging studies (Georgiewa et al., 2002; Landi et al., 2010; Wimmer et al., 2010).

In addition, in a broader sense, our results agree with those of Pugh et al. (2000), a study in which they used phonological processing tasks, orthographic processing tasks, and tasks combining both cognitive processes. Their results suggest that dyslexic subjects showed a weaker connectivity pattern than the pattern shown by normal subjects when they solved the task implying both phonological and orthographic decisions.

The DCM results of our study show an effective connectivity pattern quite different from the SEM connectivity pattern. Firstly, in the spelling recognition task in the HSS group, for whom the task is easier due to their probable visual word processing expertise, the DCM model was not estimated due to the fact that only one ROI presented statistically significant activations.

Conversely, in the LSS group, we found significant connectivity patterns between the LMFG, the RMTG, and the LITG toward the RSFG. However, as was the case in the effective connectivity analysis through SEM, the intensity of these effects as well as the intensity of each ROI’s self-activation with itself is small.

These data suggest again that, in both groups, reading is a globally different process in terms of brain activation. The impossibility to estimate DCM in the HSS group is due to the fact that only one ROI reaches statistical activation and in consequence it is impossible to estimate connectivity models.

This situation means that – for this group – there are a smaller number of activated clusters that can explain a statistical model. Strictly speaking, there are no other statistical effects other than the self-correlation effects for this specific ROI. It might be thought that the subjects with high skills found so little difficulty in the spelling task that they did not need special connectivity networks to meet the demand. This would be consistent with some previous results by Zarabozo-Hurtado et al. (under revision) showing similar effects in the estimation of simple effects. The lack of papers on connectivity in this type of task for this specific population prevents us from delving further into the discussion of this aspect.

Conversely, in the LSS group, the DCM pattern is more complex than in the HSS group. In fact, these results are congruent with those found by Saur et al. (2010).

Some studies suggest that individuals with reading problems present a series of compensatory mechanisms at brain level when facing the complexity it means for them to execute this cognitive task (Sandak et al., 2004; Gebauer et al., 2012). These data support our results. The DCM results of the visuoperceptual recognition task show that, in the LSS group, there is only one significant effect of the left pre-central gyrus toward the RSFG. The group of good readers, instead, presents a more complex effective connectivity pattern, where the precentral gyrus of both hemispheres receives the majority of the effects, although they are of low intensity. This could represent that the HSS group is consistently mapping between orthography and phonology, even though orthographic recognition was not requested in this task.

The fact that it is the subjects from the HSS group presenting the more complex DCM model, however, is not as surprising as it might seem. The execution of a relatively simple task, such as finding a letter in a word, which was executed correctly by the low-spelling skills group, might be affected by the automatization of the orthographic processing of words, where the presence of orthographic errors increases the task’s difficulty only for those subjects who have developed a specialization in recognizing orthographic patterns, like the HSS group had.

However, in adults with low orthographic abilities, an orthographic violation is not automatically processed, probably due to weaker orthographic representations in long term memory or to a poorer development of the orthographic lexicon. In other words, the subjects from the HSS group, in the C-D pair of blocks (visuoperceptual recognition task) would be conducting two tasks at once: vowel detection, as requested, and, involuntarily or automatically, orthographic mapping.

Despite the above comments in relation to HSS group, it is important to bear in mind the small sample size used to estimate the statistical effects. There are several DCM models estimated with small sample sizes, but there is not enough evidence about the effects of sample size on the connectivity modelization process (Benetti et al., 2009; Sato et al., 2009; Rowe, 2010).

Our paper presents some limitations that need to be discussed. The main limitation, in our opinion, is the fact that the subjects were selected among students in the senior year of high school and, in light of their reading performance, some subjects from the LSS group might have been dyslexic but, as far as we know, none of them had been diagnosed previously. In other words, some of the participants from this group might have suffered from a relatively mild form of dyslexia that would have been compensated by their own means allowing them to reach the senior year. On the other hand, no measurement or estimation instrument was applied to them for intellect, which might have also influenced their performance in this study’s tasks.

Nonetheless, these limitations need to be clarified. Firstly, the orthographic abilities tests used to form the groups were very thorough, which allowed us to form the HSS and LSS groups with wide knowledge of the subjects’ reading performance at the moment of inclusion in the study, and it also allowed us to have much intra-group homogeneity as regards their current reading skills. Additionally, the fact that all the subjects, both HSS and LSS, were students from the same degree of high school makes it unlikely that there were great differences in the general intellectual functioning of both groups, which makes our results hardly questionable in this sense.

We would also like to note that, to conduct this study, a 1.5T scanner was used with a TR of 3. There is a possibility that, with a 3T scanner, the DCM model could be estimated for the HSS group in the spelling recognition task. However, our data suggest that, in that case, our results would point in the same direction, that is, a very simple DCM model in the HSS group when compared to the LSS group, thus suggesting that the task is easy for these subjects. Still, even if these conditions are not ideal for connectivity studies, recent studies on effective connectivity have used similar equipment to the one used in the present study (Hildebrant et al., 2013).

Another limitation of our study is the sample size we selected, which may be considered rather small. However, this should be seen as a relative limitation. The criteria to confirm the groups were strict, and the method of assignment to the groups, following the extreme values technique, allowed us to maximize the possible differences. This made data interpretation rather clear in terms of brain activation despite the relatively small sample size (Friston, 1998, 2012; Logothetis, 2002). Apart from the sample size, the regularity of the effects and the activations found in the intra-group effects guarantee the homogeneity of the sampling and the correct application of the experimental procedure. In any case, it might be important to dedicate some efforts through simulation procedures to estimate the impact and effect of the sample size on the estimation of models for connectivity, SEM and DCM.

Our paper also has some strengths that deserve comment. The most remarkable one is that this is, to our knowledge, the first paper exploring the effective connectivity model, estimated through SEM, and the efficient connectivity model, estimated through DCM, in a visual recognition task of homophone errors in Spanish, while at the same time controlling the subjects’ level of orthographic competence and, consequently, separating those with a high level from those with a low level of competence. As has been commented above, this type of error is characteristic of transparent languages, especially of the variety of Spanish spoken in different parts of Latin America. In this sense, our results are particularly interesting, given that this type of orthographic errors is characteristic and very usual of transparent languages, where reading as a cognitive function has some distinctive features.

More studies should be analyzed to see whether the activation patterns observed in this study are found when facing detection tasks of other types of orthographic errors, or, on the contrary, homophone error detection activates a pattern in good and bad readers somewhat different from other types of errors. In the future, we should obtain more detailed information about brain activities in order to analyze more statistically complex models like Farràs et al. (under revision) suggest.

To conclude, we can say that taken globally, the analyses of the connectivity of the tasks under study through SEM and through DCM present some similarities. The first one is that both the SEM and the DCM models show distinctive connectivity patterns between the HSS and LSS groups. Likewise, both types of analyses suggest patterns with effective connectivity in one case (SEM) and the other (DCM) that are much clearer and with more intense effects in the case of the HSS group as compared to the LSS group. This is much clearer, obviously, in the case of the visuoperceptual recognition task.

Nonetheless, they also present important differences. The DCM models are very different between both groups under study, for the spelling and visuoperceptual recognition tasks. In fact, the DCM models probably reflect somewhat better what happens with the behavioral conduct of the tasks under study. A clear interaction effect was revealed between group and task, so that the HSS group conducted the spelling recognition task more efficiently than the LSS group. Likewise, as we commented above, in the visuoperceptual recognition task, the effective connectivity pattern was more complex in the HSS group than in the LSS group. However, it was due to the fact that the subjects who are good readers probably carry out both tasks at the same time, whereas the LSS subjects would only carry out the visuoperceptual vowel recognition task, their reading being much less automatized than that of the good readers.

Furthermore, we consider it important to remark that it is essential to continue with this research line. It might be interesting to analyze the ROIs that emerge when we request the subjects to conduct an automatic process by differentiating HSS and LSS groups, like the Stroop task. Another important line to continue is the analysis of people who have in fact a real orthographic problem, for example working with dyslexic persons as compared to persons with good competences in orthography.

Finally, these data point in two complementary directions for future research. Firstly, we should approach the estimation of connectivity models when faced with these tasks or similar ones with samples from the same populations but with a larger amount of ROIs, not just the ones generated from data-driven approaches. Instead, more theoretical models should be analyzed and their possibilities of empirical and statistical evidence evaluated for viability. Secondly, we must work in a more structured way on the analysis of limitations and possible improvements of the statistical models we use to estimate connectivity since they involve a special conception of the way connectivity works and it entails a specific way to understand that complex reality.

## Conflict of Interest Statement

The authors declare that the research was conducted in the absence of any commercial or financial relationships that could be construed as a potential conflict of interest.
